# Prolonged versus single dose in penicillin oral challenge testing: protocols for a pilot and definitive randomised controlled trial (PROSPECTOR studies)

**DOI:** 10.1136/bmjopen-2024-094712

**Published:** 2025-02-22

**Authors:** Irvin Ng, Fiona James, Ana Copaescu, Sara Vogrin, Elise Mitri, Morgan Rose, Richard Sullivan, Michael Lane, Amy Legg, Jack Godsell, Suran Fernando, Lene Heise Garvey, Vito Sabato, Philip Li, Jonathan Grant Peter, Jason Trubiano

**Affiliations:** 1 Infectious Diseases, The Peter Doherty Institute for Infection and Immunity, Melbourne, Victoria, Australia; 2 Infectious Diseases and Immunology, Austin Health, Heidelberg, Victoria, Australia; 3 National Allergy Centre of Excellence, Parkville, Victoria, Australia; 4 Peter MacCallum Cancer Centre, Melbourne, Victoria, Australia; 5 Infectious Diseases, St George Hospital, Kogarah, New South Wales, Australia; 6 Royal Brisbane and Women’s Hospital, Herston, Queensland, Australia; 7 Pharmacy Department, Royal Brisbane and Women’s Hospital, Herston, Queensland, Australia; 8 Allergy and Immunology, The Royal Melbourne Hospital, Parkville, Victoria, Australia; 9 Clinical Immunology and Allergy, Royal North Shore Hospital, St Leonards, New South Wales, Australia; 10 University of Copenhagen, Kobenhavn, Region Hovedstaden, Denmark; 11 Allergy Clinic, Department of Dermatology and Allergy, Herlev and Gentofte Hospital, Gentofte, Denmark; 12 Department of Immunology, Allergology and Rheumatology, University of Antwerp, Antwerpen, Belgium; 13 Department of Immunology, Allergology and Rheumatology, University Hospital Antwerp, Edegem, Antwerp, Belgium; 14 Division of Rheumatology and Clinical Immunology, Department of Medicine, Hong Kong University, Hong Kong, Hong Kong; 15 Division of Allergy and Immunology, Department of Medicine, University of Cape Town, Cape Town, South Africa

**Keywords:** Clinical Trial, Antibiotics, PUBLIC HEALTH, Protocols & guidelines, IMMUNOLOGY

## Abstract

**Introduction:**

Penicillin allergy labels (PALs) are reported in 1 in 10 hospitalised patients globally and associated with inferior patient, hospital and microbiological outcomes; however, the majority are incorrect and should be removed. Direct oral penicillin challenge has been demonstrated to be a safe and effective method for the removal of PALs. However, the question of whether a single dose is sufficient to ascertain true allergy status remains unanswered, with some studies suggesting that extended challenges of 3 or more days are superior for the exclusion of delayed immune reactions. The aim of the PROSPECTOR studies was to determine the feasibility (PROSPECTOR-1) of a definitive trial (PROSPECTOR-2) to evaluate the safety and effectiveness of prolonged oral challenge (ie, 5 days) versus single-dose oral challenge in patients with a delayed or unknown penicillin allergy phenotype (PROSPECTOR-2).

**Methods and analysis:**

A pair of double-blind two-arm parallel placebo-controlled trials will be undertaken**—PRO**longed versus **S**ingle dose in **PE**nicillin oral **C**hallenge **T**esting double-blind parallel group randomised placebo-c**O**ntrolled t**R**ial (PROSPECTOR Studies). Patients with a reported delayed or unknown timing penicillin allergy who have passed a supervised single-dose oral amoxicillin challenge (with or without prior skin testing/single or split dose) will be recruited. Informed patient consent will be granted for sites to recruit patients and collect routine clinical data. PROSPECTOR-1 will assess the safety and feasibility of a placebo-controlled trial for single-dose amoxicillin challenge versus 5-day prolonged oral challenge. PROSPECTOR-2 will assess the superiority of the 5-day prolonged oral challenge compared with single-dose amoxicillin challenge in excluding a delayed immune reaction. PROSPECTOR-2 will commence immediately post completion of PROSPECTOR-1 in a vanguard design, with adjustments to the projected sample size for superiority made following completion of PROSPECTOR-1. PROSPECTOR-2 will commence recruitment immediately following closure of PROSPECTOR-1; however, data from each trial will be analysed separately.

**Ethics and dissemination:**

These studies were reviewed and approved by the Austin Health Human Research Ethics Committee (PROSPECTOR-1: HREC/99740/Austin-2023 and PROSPECTOR-2: HREC/109785/Austin-2024). The results will be published in peer-reviewed journals and presented at relevant conferences.

**Trial registration number:**

PROSPECTOR-1: ACTRN12623001242617 and PROSPECTOR-2: ACTRN12624001107516.

**STRENGTHS AND LIMITATIONS OF THIS STUDY**

- These studies are among the first double-blind randomised placebo-controlled trials in antibiotic allergy investigation.
- The pilot phase, randomised experimental design and recruitment of patients from existing inpatient or outpatient settings will minimise the opportunity for selection bias.
- The definitive trial is international and multicentre, allowing for increased sample heterogeneity and generalisability of results.
- The pilot study does not aim to explore the ideal number of days for prolonged challenge in eliciting true delayed allergy, so conclusions will be limited to comparison with a prolonged 5-day challenge, while there is still variability in practice globally.
- A two times a day 500 mg dose of penicillin is set as the intervention; however, variability in preferred dosage remains in prolonged challenge practice globally.

## Introduction

### Background and rationale

 Penicillin allergy labels (PALs) are commonly documented in patient electronic medical records (EMRs).^1^ At the higher end, the prevalence has been estimated at 10% for hospitalised Australians^2^ and 9.9% of inpatients in Montreal, Canada.^3^ A Danish study found that 5% of hospital inpatients carried a PAL,^4^ while at the lower end, the prevalence is estimated at 3.2% in hospital inpatients in South Africa^5^ and 2% for all beta-lactam allergy in Hong Kong patients.^6^ This figure may be even higher among vulnerable patients such as the immunocompromised,^7^ geriatric and rheumatology populations.^8 9^ Those patients who carry a PAL are more likely to receive an inappropriate antibiotic, suffer a hospital-associated adverse event and acquire a multidrug-resistant organism.^10^,^13^ PALs are also associated with increased hospital length of stay (LOS), higher readmission rates, increased hospital costs and mortality rates.^10 14 15^ At a public health level, they are associated with inappropriate prescribing and antimicrobial resistance.^1^

Despite their omnipresence, the majority of PALs are assessed as ‘low-risk’ and can be safely removed by penicillin allergy testing.^16^,^19^ Oral penicillin challenge with or without preceding skin testing is considered the gold standard for delabelling^20^; however, clinical equipoise remains regarding the superiority of single-dose or prolonged (ie, multiple-day dosing) oral challenge for patients who report a delayed or unknown timing penicillin allergy phenotype. The current Drug Allergy Practice Parameters recommend ‘against the routine use of multiple-day challenges in the evaluation of penicillin allergy’, providing a ‘strong recommendation’ but with ‘low certainty of evidence’.^21^ The European guidelines reviewed the literature of over 6484 patients, demonstrating a 2.3% positive rate following the initial challenge and 5.5% during the varied prolonged challenges. They concluded that there is no consensus on a preferred procedure and could not provide a recommendation for or against prolonged oral challenge.^22^

The results from a mixture of European observational and retrospective studies suggest that prolonged challenges ranging from 3 to 10 days may be superior to single-dose challenges at eliciting delayed immune reactions. However, the reported prevalence of delayed reactions is highly variable (5–12% of patients), and many were reliant on patient self-reporting.^23^,^30^ In a recent retrospective single-centre Danish study of 3179 low-risk patients, 2.6% were positive on day 1 of challenge and 7.2% after day 1.^31^ This contrasts with the North American experience, where prolonged challenges have been associated with low rates of delayed reactions (0–1.8%).^32^,^35^ A paediatric study demonstrated that delayed reactions may occur <7 days following a single challenge.^36^ This gap between guideline recommendations and evidence, and differing results across geographical regions, highlights the need for robust evidence to inform practice with clinical certainty.

Randomised controlled trials (RCTs) have not routinely been used to answer questions regarding best practice in penicillin allergy research, despite their ability to provide high-quality evidence. The PROSPECTOR (**PRO**longed versus **S**ingle dose in **PE**nicillin oral **C**hallenge **T**esting double-blind parallel-group randomised placebo-c**O**ntrolled t**R**ial) studies will use a double-blind, parallel-group, placebo-controlled RCT study design. PROSPECTOR-1 is an external pilot trial, which will assess the feasibility of conducting a blinded placebo-controlled trial of single-dose versus prolonged dose oral challenge in patients with a documented or reported PAL. It will also inform the sample size of a definitive full-scale trial, which will follow directly on from PROSPECTOR-1, with adjustments made as required based on effectiveness and feasibility outcome data. PROSPECTOR-2 is the definitive trial, which will assess whether a prolonged oral challenge is superior to a single dose challenge for ascertaining true immune-mediated penicillin allergy.

### Objectives

PROSPECTOR-1: To evaluate the feasibility of a placebo-controlled trial and inform the design of a definitive trial evaluating whether prolonged oral challenge (5 days) is superior to single-dose oral challenge in patients reporting penicillin allergy with delayed or unknown timing phenotype to ascertain a true immune-mediated adverse reaction. PROSPECTOR-2: To evaluate the effectiveness of prolonged oral penicillin challenge (5 days) over single-dose penicillin challenge for ascertainment of true penicillin allergy (ie, immune-mediated allergy).

### Trial design

The PROSPECTOR studies are multicentre, prospective, double-blinded, placebo-controlled, parallel-group, randomised controlled trials: PROSPECTOR-1 is an Australian external pilot study for a future definitive, international, Phase III trial, PROSPECTOR-2.

### Methods: participants, interventions and outcomes

**Figure 1:**
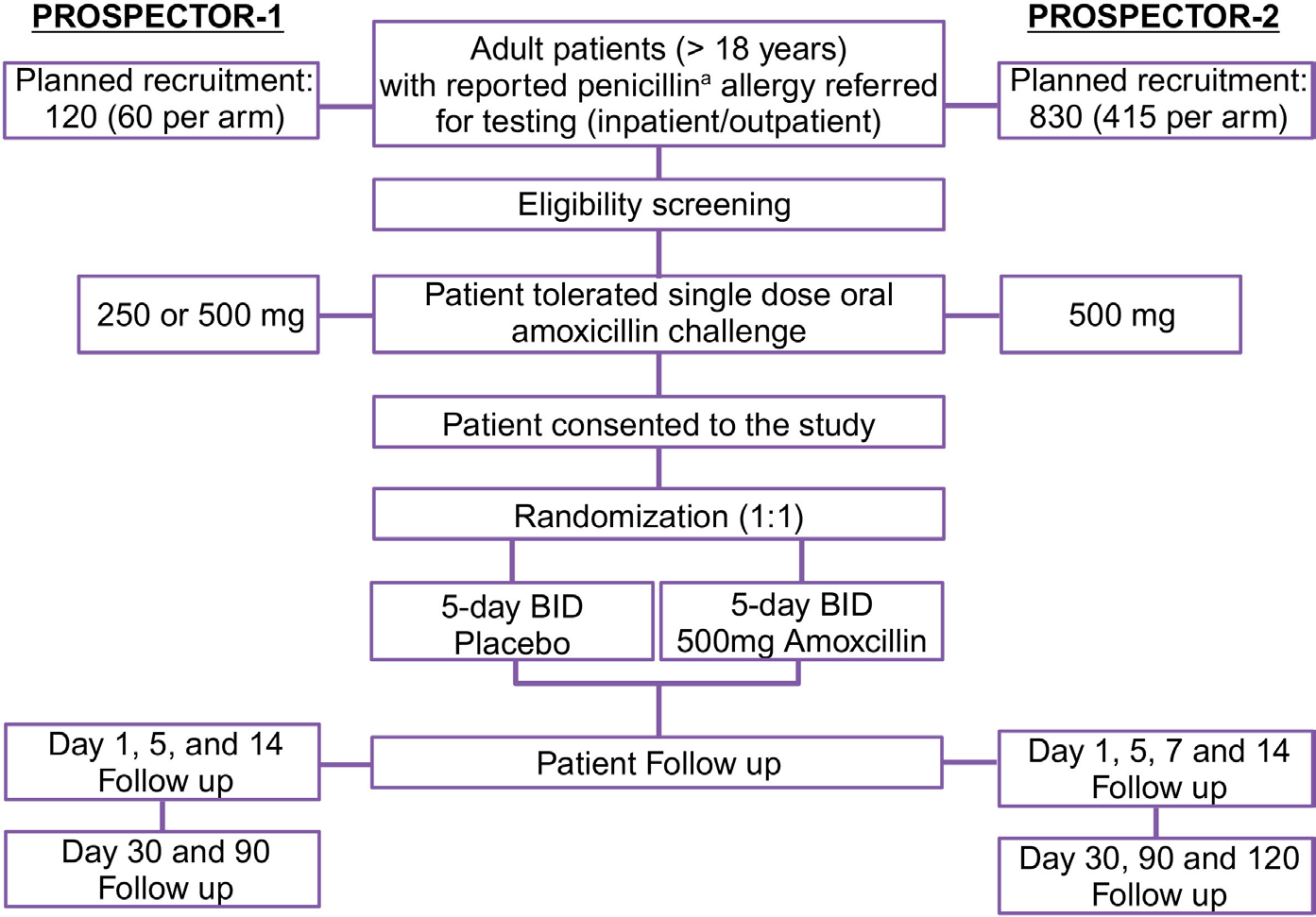
Study design for PROSPECTOR-1 (**PRO**longed versus **S**ingle dose in **PE**nicillin oral **C**hallenge **T**esting double-blind parallel-group randomised placebo-c**O**ntrolled t**R**ial) and PROSPECTOR-2 studies. ^a^penicillin ‘unspecified’, penicillin VK, penicillin G, amoxicillin, ampicillin, flucloxacillin, dicloxacillin, cloxacillin, mecillinam, pivmecillinam and pivampicillin. BID, two times a day.

The study design for PROSPECTOR-1 and PROSPECTOR-2 is outlined in figure 1.

#### Study setting

PROSPECTOR-1 will be undertaken at four tertiary hospital centres in Australia, including Austin Health (Victoria), Peter MacCallum Cancer Centre (Victoria), St George Hospital (New South Wales) and Royal Brisbane and Women’s Hospital (Queensland). PROSPECTOR-2 will be expanded to 14 tertiary hospital centres across Australia, Asia, North America, Africa and Europe (a complete list may be viewed in online supplemental table 1).

#### Eligibility criteria

Adult patients referred to inpatient or outpatient allergy services for a suspected immune-mediated penicillin allergy with a history of delayed or unknown timing will be eligible for inclusion in the study. Participants will then be risk-assessed using the PEN-FAST tool^37^ and RegiSCAR score. Participants will receive a single dose of 250–500 mg (PROSPECTOR-1) or 500 mg (PROSPECTOR-2) amoxicillin challenge (with or without prior skin testing). Participants who pass this initial challenge without an observed immune-mediated adverse event (1–2 hours post dose) will proceed to randomisation. The inclusion and exclusion criteria are listed in box 1.

Box 1 **Inclusion and exclusion criteria**

**Inclusion criteria**

- Adult patients referred to the inpatient or outpatient allergy services for a suspected penicillin allergy with an immune-related allergy history of delayed (>6 hours after first dose of drug administration) or unknown timing, who tolerate the first single dose of an oral amoxicillin challenge.
- Willing and able to give consent and undergo telehealth/telephone review.

**Exclusion criteria**

- The patient’s age is <18 years.
- Any other illness that, in the investigator’s judgement, will substantially increase the risk associated with the subject’s participation in this study.
- Stevens-Johnson syndrome or toxic epidermal necrolysis to beta-lactam.
- Inpatients concurrently receiving or likely to receive a beta-lactam antibiotic therapy during the 14-day study period.
- The concurrent use of antihistamines and systemic steroid therapy (ie, >10 mg daily) (PROSPECTOR-2).

### Interventions

#### Intervention

Participants randomised to the intervention arm will receive a prolonged, 5-day course of oral 500 mg amoxicillin, administered two times a day, to commence the day following the initial single-dose amoxicillin oral challenge.

#### Control

Participants randomised to the control arm will receive a prolonged, 5-day course of oral placebo (microcrystalline cellulose (MCC), prepared to be visually identical to the intervention, administered two times a day, to commence the day following the initial single-dose amoxicillin oral challenge).

#### Preparation and dispensing

A qualified pharmacy staff member will dispense the study medication in unique container numbers to a member of the investigator team. A second staff member will verify the dispensing. The participant or their caregiver should be instructed to maintain the product in the bottles provided throughout the course of dosing and return the bottles to the site at either a study visit or via a return registered post.

#### Study compliance

Participants will be asked to record the date and time of each dose of study medication using an electronic or paper diary. Compliance will be assessed at each scheduled visit by study staff who will ask the participant to count the number of capsules remaining. The following non-compliance cases will be recorded as protocol deviations:

- Study medication missed for ≥2 consecutive doses.
- Study medication compliance<80%.

### Outcomes

The primary outcomes for the PROSPECTOR studies are outlined below, with secondary outcomes listed in table 1.

**Table 1:** Secondary outcome measures for **PRO**longed versus **S**ingle dose in **PE**nicillin oral **C**hallenge **T**esting double-blind parallel-group randomised placebo-c**O**ntrolled t**R**ial (PROSPECTOR) studies

	PROSPECTOR-1	PROSPECTOR-2
Positive oral challenge	Up to day 7 following the first hospital-administered single dose	Up to day 14 following the first hospital-administered single dose
Feasibility	Recruitment rate per site (recruitment/site/month) Randomisation to recruitment ratio (n, %) Withdrawal (n, %) Loss to follow-up (n, %) Missing data Protocol compliance (n, %)	Not applicable
Safety	Severe adverse reaction (n, %) Immune-mediated adverse event or severe adverse drug reaction (n, %) Non-immune-mediated adverse event (n, %) Any cutaneous adverse reaction (n, %)	Immediate severe adverse reaction (anaphylaxis or death) (n, %) Delayed adverse reaction (severe cutaneous adverse reaction) (n, %) Non-immune-mediated adverse event (n, %) Grade three or four adverse reactions as defined by World Allergy Organization (ref: Sanchez-Borges *et al*, 2019) (n, %) Any cutaneous adverse reactions (n, %)
Efficacy	*Clostridioides difficile* infection at days 30 and 90 (n, %) Isolation of a multidrug-resistant infection at days 30 and 90 (n, %)	*C. difficile* infection at days 30, 90 and 120 (n, %) Multidrug-resistant infection at days 30, 90 and 120 (n, %) Multidrug-resistant colonisation at days 30, 90 and 120 (n, %)
Cost-effectiveness	Cost-effectiveness of placebo vs open-label trial	Cost-effectiveness analysis of prolonged vs single-dose oral challenge
Quality of life	Not applicable	Health-related quality of life outcome, measured by shortened Drug Hypersensitivity Quality of Life Questionnaire (online supplemental table 3)^43^ at day 0 and day 90.

#### PROSPECTOR-1

Compliance with the intervention (proportion of participants (n, %) taking at least 80% of the doses), need for unblinding (proportion of participants (n, %) being intentionally or unintentionally unblinded) and recruitment to eligibility ratio (proportion of participants (n, %) consented to the study from eligible participants). Subgroup analyses for admission setting (inpatient vs outpatient), risk (PEN-FAST score <3 vs ≥3) and severity (RegiSCAR score <2 vs ≥ 2) will be performed in the PROSPECTOR-1 study.

#### PROSPECTOR-2

The proportion of positive oral challenges (ie, immune-mediated reaction up to and including day 7 following the first test dose, as adjudged by an independent blinded panel), n (%). Subgroup analyses for the following parameters: admission setting (inpatient vs outpatient), index reaction phenotype—delayed versus unknown timing, risk (PEN-FAST score 0 vs 1–2 vs ≥3), immunocompromised status, sex, region (Australia vs Europe vs Asia vs North America vs Africa), clinic type (specialised allergy clinic vs non-allergy clinic), index reaction severity (RegiSCAR <2 vs ≥2), index reaction phenotype—severe MPE (RegiSCAR score 1 or 2) versus RegiSCAR score <1 versus RegiSCAR score >2 and index reaction timing—delayed exanthema <5 years post index reaction versus delayed exanthema >5 years post index reaction.

### Participant timeline

The participant timeline is outlined in a schedule of enrolment, interventions and assessments for both studies (online supplemental table 2). After randomisation on day 0, participants will be provided with the study medication and a telehealth review will be scheduled for days 1, 5, 7 and 14 by a specialist allergy healthcare provider (ie, board-certified allergist, clinical immunologist and other clinicians with specialised training in allergy and immunology). If patients are inpatients at the time, then this review will be performed at the patient bedside. At each telehealth review, compliance will be recorded by reporting the number of doses taken, and participants will be asked about any other concurrent antibiotic therapy. If a positive oral challenge is reported, a summary of the patient-reported symptoms using a standardised questionnaire and clinical photography of any rash, cutaneous or mucosal changes will be sent to an independent review panel consisting of an allergist and dermatologist blinded to the intervention to ascertain if the reported reaction is an ‘immune-mediated adverse drug reaction’.

At days 30 and 90 post-randomisation, a telephone questionnaire and assessment of the medical record will be undertaken to assess for secondary outcomes including antibiotic-associated diarrhoea, *Clostridioides difficile* infection or acquisition of a multidrug-resistant organism. Patients at the day 90 follow-up will be unblinded if preferred by the site principal investigator, and those in the control arm will be offered a prolonged oral challenge.

### Sample size

PROSPECTOR-1: A total of 120 participants are planned for inclusion (60 per arm). This sample size was chosen to provide a precise estimate of feasibility outcomes with a width of CI being <20% for any proportion. Such a sample size would also likely provide a reliable estimate of effectiveness as it has been shown that with binary outcomes, gain in precision is smaller once each group reaches 60 participants.^38^ This sample size also likely represents >9% of the definitive trial’s sample size (to detect a 5% difference assuming 8% event rate with 90% power and 5% significance level, a total of almost 900 participants would be required).^38^

PROSPECTOR-2: A total of 830 participants are planned for inclusion (415 per arm). The incidence of delayed reactions after single-dose oral challenge in the current literature is approximately 3%,^20^ while the incidence of delayed reactions after prolonged oral challenge is approximately 8%.^27 39^ To detect a 5% difference with 85% power and 5% significance level, 372 participants would need to be randomised to each arm. To account for a 10% loss to follow-up, a total of 830 participants will be recruited.

As the stipulated 5% difference reported in the literature and observed in our pilot data is not regarded as clinically relevant by many drug allergy specialists, we will also evaluate the non-inferiority of single-dose challenge. Given the lesser severity of an adverse event, a clinically relevant non-inferiority margin was determined to be 10% among investigators of this study. The planned sample size will enable us to evaluate a non-inferiority of the risk difference between study arms (secondary outcome) with double-sided 95% CI with 82% power (assuming real difference between arms being 5%).^40^ The sample size for PROSPECTOR-2 will be updated prior to study start based on the estimates observed in PROSPECTOR-1.

### Recruitment

Recruitment will be undertaken by appropriately trained and delegated study investigators at participating sites in both the ambulatory clinic and inpatient settings. The central study team will monitor and encourage recruitment by regularly engaging with participating site staff, providing strategies for boosting enrolment and troubleshooting solutions. Recruitment number updates will be communicated through a regular study newsletter.

### Consent

Eligible patients will be provided with a verbal explanation of the project by a delegated study investigator and a paper or electronic consent form to read through (online supplemental materials 1 and 2). They will be encouraged to ask questions and discuss their participation with family, friends or a trusted family doctor if helpful. A thorough assessment of the participant’s capacity to make a valid informed decision will be made by the study investigator before the patient is recruited and documented informed consent is obtained.

### Methods: assignment of interventions

#### Allocation

Permuted block design randomisation will be used, stratified by the hospital site and setting (inpatient vs outpatient). While block design might result in larger treatment imbalances, such design is preferred to overcome logistical difficulties. Randomisation will be performed by the unblinded pharmacy dispensing team via REDCap just before the intervention. The allocation sequence will be concealed until the time of the randomisation.

#### Blinding

Participants (and their caregivers if applicable) as well as study investigators and research staff will be blinded to the assigned intervention. Adverse Event Review Panel members will be blinded to the assigned intervention. Clinical trials pharmacists will be unblinded.

Participants may be unblinded during the 7-day follow-up period in the event of a serious adverse event (SAE) or grades 3 or 4 adverse event and if the site principal investigator deems this appropriate. If a participant’s assignment is revealed, the Sponsor and Coordinating Principal Investigator will be notified within 24 hours of unblinding. The date and reason for the blind broken must be recorded in the source documentation and case report form. All participants may be unblinded at the 90-day follow-up to allow for those in the control arm to receive a prolonged challenge if that is the site’s practice or preference.

### Methods: data collection, management and analysis

#### Data collection methods

De-identified clinical data will be stored in a secure electronic REDCap database, hosted by the University of Melbourne. Each participating centre will only have access to their own patient data. All electronic and paper data will be retained for a period of 15 years after which all data will be destroyed according to hospital policy in place at the time.

#### Progression from pilot to definitive trial

PROSPECTOR-2 will proceed immediately on completion of PROSPECTOR-1, provided the following criteria are met:

1. Compliance with study medication is ≥80%.
2. Unblinding is ≤10%.
3. Recruitment to eligibility ratio ≥80%.

If these criteria are not met, appropriate amendments to the study design of PROSPECTOR-2 will be made. If compliance is under 80%, additional reminders will be scheduled for participants. If unblinding exceeds 10%, the trial will be converted to open label. If the recruitment to eligibility ratio is lower than 80%, additional strategies for recruitment may be considered.

### Statistical methods

#### PROSPECTOR-1

The results will be presented according to CONSORT guidelines for feasibility studies.^41^ Patient characteristics and penicillin allergy history will be presented by arm using median (IQR) for continuous variables and count (percentage) for categorical variables. Binary outcomes will be presented as count and percentage with 95% exact CIs. All outcomes (where feasible) will be presented as overall, by study arm and by setting. Exploratory efficacy outcomes will also be presented as absolute (risk difference) and relative difference (risk ratio) with 95% CIs. No statistical tests will be performed. The amount and pattern of missing data will be explored.

#### PROSPECTOR 2

The results will be presented according to CONSORT guidelines.^42^ Patient characteristics and penicillin allergy history will be presented by arm using median (IQR) for continuous variables and count (percentage) for categorical variables. The primary analysis will be on an intention-to-treat basis. A generalised linear model with binomial family will be used to calculate the risk difference (identity link) and risk ratio (log link) between intervention and control. The results will be presented with two-sided 95% CIs. Models will be adjusted for stratification variables (clinical site and setting). Subgroup analysis will be performed by the inclusion of an interaction term between subgroup and arm. The primary analysis will also be performed in the per-protocol population. Time to adverse reaction will be evaluated using the Kaplan-Meier method and Cox proportional hazards regression. A detailed statistical analysis plan will be prepared and uploaded to the Australian New Zealand Clinical Trials Registry (ANZCTR) listing before study completion.

### Oversight and monitoring

#### Adverse event review panel

A blinded independent review panel consisting of an allergist and a dermatologist will be established to review reported adverse drug reactions for both PROSPECTOR studies. A summary of the patient’s reaction will be compiled, comprising a standardised symptom questionnaire and clinical photography of any rash, cutaneous or mucosal change. The review panel will provide a determination of whether the reported reaction is to be classified as an ‘immune-mediated adverse drug reaction’. This classification will be provided to the data safety monitoring board (DSMB) for further deliberation in addition to the stipulated reports.

#### Data safety monitoring board

A DSMB will be established to review trial data and monitor the progress of each trial. The DSMB will monitor adherence to the protocol, participant recruitment, outcomes and participant safety data. They will also monitor the assumptions underlying sample size calculations for the study and alert the investigators if an increased recruitment effort is required. The DSMB will make recommendations as to whether the study should continue or be terminated, consider participant safety or other circumstances as grounds for early termination, including either compelling internal or external evidence of treatment differences or feasibility of addressing the study hypotheses (eg, poor participant enrolment).

## Ethics and dissemination

These studies were reviewed and approved by the Austin Health Human Research Ethics Committee (Reference Numbers: PROSPECTOR-1: HREC/99740/Austin-2023 and PROSPECTOR-2: HREC/109785/Austin-2024). Additional approvals will be sought for international PROSPECTOR-2 sites before their participation in the study. The results will be published in peer-reviewed journals and presented at relevant conferences.

### Harms

Adverse events will be recorded from the time of randomisation until day 90. Patients will be supplied with a prescription for oral corticosteroids and second-generation antihistamines on hospital discharge, only to be used in the event of an immune-mediated positive oral challenge, as instructed by the site investigators at the time of the days 1, 5, 7 and 14 reviews. Participants will be instructed by site investigators to fill this script at their own expense if required.

### Patient and public involvement

No patient and/or public were involved in the study development.

## supplementary material

10.1136/bmjopen-2024-094712 online supplemental file 1
