# Supplementary material for: Prolonged versus single dose in penicillin oral challenge testing: protocols for a pilot and definitive randomised controlled trial (PROSPECTOR studies)
Source: BMJ Open. 2025 Feb 22;15(2):e094712. doi: 10.1136/bmjopen-2024-094712 (PMC11848670; doi:10.1136/bmjopen-2024-094712)
Supplement: online supplemental file 1 [file bmjopen-15-2-s001.docx]

**SUPPLEMENTARY Materials**

**Supplementary Table 1.** List of study centres for PROSPECTOR-1 and PROSPECTOR-2

| Site | Address | Site PI | PROSPECTOR-1 | PROSPECTOR-2 |
| --- | --- | --- | --- | --- |
| Austin Health | 145 Studley Road, Heidelberg VIC 3084, Australia | Prof Jason Trubiano | X | X |
| Peter MacCallum Cancer Centre | 305 Grattan Street, Melbourne, VIC 3000, Australia | Dr Morgan Rose | X | X |
| Royal Melbourne Hospital | 300 Grattan Street, Parkville VIC 3052, Australia | Dr Jack Godsell |  | X |
| St George Hospital | Gray Street, Kogarah, NSW 2217, Australia | Dr Richard Sullivan | X | X |
| Royal North Shore Hospital | Reserve Road, St Leonards NSW 2065, Australia | Prof Suran Fernando |  | X |
| Royal Brisbane and Women’s Hospital | Butterfield St, Herston QLD 4006, Australia | Dr Michael Lane | X | X |
| Royal Adelaide Hospital | Port Rd, Adelaide SA 5000, Australia | A/Prof William Smith |  | X |
| Sir Charles Gairdner Hospital | Hospital Ave, Nedlands WA 6009, Australia | Prof Michaela Lucas |  | X |
| Montreal General Hospital | 1650 Cedar Ave, Montreal, Quebec H3G 1A4, Canada | Dr Ana Copaescu |  | X |
| Allergy Clinic, Herlev and Gentofte Hospital | Gentofte Hospitalsvej 8 1. Floor, 2900 Hellerup, Denmark | Prof Lene H Garvey |  | X |
| Groot Schuur Hospital | Main Road, Observatory, Cape Town, 7935, South Africa | Prof Jonathan Peter |  | X |
| Queen Mary Hospital, University of Hong Kong | Pokfulam Road, Pokfulam, Hong Kong | Dr Philip Li |  | X |
| Antwerp University Hospital | Drie Eikenstraat 655, 2650 Edegem, Belgium | Prof Vito Sabato |  | X |

**Supplementary Table 2.** Drug Hypersensitivity Quality of Life Questionnaire (Mak HWF et al, JACI: In Pract 2024)

**Table 3**. Schedule of enrolment, interventions and assessments

|  | Screening/Enrolment* | Treatment period | | Follow up period | | | | | Notes |
| --- | --- | --- | --- | --- | --- | --- | --- | --- | --- |
|  |  | Day 1 | Day 5 | Day 7 | Day 14 | Day 30 | Day 90 | Day 120 |  |
| **Visit window** | Day -5 to 0 | (0) | (+/-1) | (+/-1) | (+/- 1) | (+/-3) | (+/-3) | (+/-3) |  |
| Informed consent | X |  |  |  |  |  |  |  | Informed consent must occur prior to all study activities |
| Baseline demographics | X |  |  |  |  |  |  |  |  |
| Baseline medical and allergy history | X |  |  |  |  |  |  |  |  |
| Concomitant medications | X | X | X | X | X |  |  |  | Record any new medications started during the study period or within 5 days of enrolment |
| DrHy-Q Questionnaire | X |  |  |  |  |  | X |  |  |
| Vital signs measurement | X | X |  |  |  |  |  |  | Vital signs to be measured at baseline on Day 0 (prior to single dose oral challenge) and post-dose (frequency at investigator’s discretion) |
| Single dose oral penicillin challenge | X |  |  |  |  |  |  |  | Challenge and 1-hour post-dose observation period to be completed prior to randomisation |
| Review of eligibility criteria | X |  |  |  |  |  |  |  | Review of eligibility criteria must be performed prior to randomisation |
| Randomisation | X |  |  |  |  |  |  |  | Day 0 |
| Dispense study medication | X |  |  |  |  |  |  |  | Study medication is dispensed on the day of randomisation (Day 0) |
| Study drug administration |  | Day 1 – 5  (10 doses total) | |  |  |  |  |  | Participant to self-administer study drug twice daily starting Day 1 |
| Participant completes dosing log |  | Day 1 – 5 | |  |  |  |  |  | Participant to complete dosing log. Note: If dose 1 is administered in the evening or PM of Day 1, treatment will continue until Day 6. |
| Study drug compliance check |  | X | X |  |  |  |  |  | Site research staff to perform compliance check and review dosing log |
| Adverse event monitoring |  | X | X | X | X | X | X | X | Site research staff to perform follow-up phone calls with participants to monitor AEs |
| Telehealth/telephone follow-up |  | X | X | X | X |  |  |  |  |
| Email outcomes survey |  |  |  |  |  | X | X | X |  |
| Retrieval of unused medications |  |  |  | X |  |  |  |  |  |

*Screening procedures may occur on Days -5 to Day 0, i.e. may be performed on the day of randomisation if preferred by site investigators

**Use of Shortened DrHY-Q  - Drug Allergy Quality of Life Questionnaire [43]

**Supplementary Table 3.** Drug Hypersensitivity Quality of Life Questionnaire (Mak HWF et al, JACI: In Pract 2024)

|  | **Not at all** | |  | **Very much** | |
| --- | --- | --- | --- | --- | --- |
|  | 0 | 1 | 2 | 3 | 4 |
| - - - 1. The problem of adverse reaction to drugs affects my life |  |  |  |  |  |
| - - - 1. The fact that I cannot use medication safely made me feel different from others |  |  |  |  |  |
| - - - 1. I feel anxious due to my problem of allergy reaction |  |  |  |  |  |
| - - - 1. I feel anguished due to my problem of allergy reaction |  |  |  |  |  |
| - - - 1. The idea of taking a medicine makes me feel anxious |  |  |  |  |  |
|  | **Total Score**: ____________________ | | | | |

**Supplementary Material 1.** PROSPECTOR-1 Patient Information and Consent Form

PATIENT INFORMATION AND CONSENT FORM

*Adult providing own consent*

| **Title** | Prolonged versus single dose in penicillin oral challenge testing randomized control trial – PROSPECTOR Study |
| --- | --- |
| **Short Title** | PROSPECTOR Study |
| **Protocol Number** | PROSPECTOR Version 7 dated 22 August 2023 |
| **Coordinating Principal Investigator** | Professor Jason A Trubiano |
| **Site Principal Investigator** | *[Principal Investigator]* |
| **Associate Investigator(s)** | *[Associate Investigator(s)]* |
| **Study Site** | *[Location]* |

# Part 1 What does my participation involve?

Between 5-15% of patients in developed countries have an allergy to penicillin (Blumenthal, 2019; Trubiano 2015). However, many of these allergies disappear over time and more than 90% are not present when tested with oral challenge (with or without prior skin testing).

Among patients reported to having a delayed onset of penicillin allergy, it is not clear whether a single oral challenge (i.e. single test dose) or a prolonged challenge (i.e. multiple day test dose) is the best in revealing one’s allergy status. In this study, participants will be randomised to either a single oral challenge given as routine clinical practice or a prolonged oral challenge (5 days).

**1 Introduction**

You are invited to take part in this research project because you have either of the following:

- a reported penicillin allergy that is delayed in onset (e.g. after 2 hours post the dose), or
- unknown in timing of allergy onset and have passed a single dose oral challenge in clinic*.*

A research personnel will ask for your allergy and medical history. Your healthcare team will guide you through a single-dose oral challenge during a clinic appointment. This will be completed as part of routine clinical practice. Only when you pass this challenge, you will be randomly assigned to:

**Group 1: Placebo**

5 days of placebo* capsules twice a day with no amoxicillin

*The placebo looks the same as an amoxicillin capsule but will not contain amoxicillin. It is a sugar-filled capsule.

**Group 2: Penicillin**

5 days of amoxicillin capsules twice a day

You will not know if you have the placebo or amoxicillin as both are in the same capsule form.

Upon discharge, you will be supplied with a prescription for oral corticosteroids and antihistamines to be used in the setting of an immune-mediated positive oral challenge. A site investigator will instruct you to fill this script at your own expense if required.

This *Participant Information Sheet/Consent Form* tells you about the research project. It explains the tests and treatments involved. Knowing what is involved will help you decide if you want to take part in the research.

Please read this information carefully. Ask questions about anything that you don’t understand or want to know more about. Before deciding whether or not you can take part, you might want to talk about it with a relative, friend or your local doctor.

Participation in this research is voluntary. If you don’t wish to take part, you don’t have to. You will receive the best possible care whether or not you participate.

If you decide you want to take part in the research project, you will be asked to sign the consent section. By signing it you are telling us that you:

• Understand what you have read;

• Consent to taking part in the research project;

• Consent to having the tests and treatments that are described;

• Consent to the use of your personal and health information as described.

You will be given a copy of this *Participant Information and Consent Form* to keep.

**2 What is the purpose of this research?**

The purpose of this study is to determine if a single dose of penicillin or prolonged course is required to accurately diagnose a penicillin allergy.

This study has been initiated by the study doctor, Prof Jason Trubiano. It is being conducted by Austin Health, Peter MacCallum Cancer Centre, St George Hospital, Royal Brisbane and Womens, Hospital McGill University Health Centre (MUHC), Groote Schuur Hospital and Herlev and Gentofte Hospital. There are no pharmaceutical or commercial sponsorships.

**3 What does participation in this research involve?**

This study will compare two ways of testing penicillin allergy in patients with a delayed allergy or unknown timing. Sometimes, we do not know which method is best for managing a condition. To find out, we need to compare different methods. In this study, we will put people into two groups in order to test two different methods. To try to make the groups the same, each participant is put into a group by chance (randomly).

Your allergy and medical history will be taken by a study investigator and a validated penicillin allergy assessment tool will be completed. If you have an identified penicillin allergy and have a negative single dose challenge (i.e. least likely to be a true allergy), you will be able to participate in this study.

If you are randomly assigned to the **“treatment” or test group** you will take a 5 day course of oral amoxicillin 500mg twice per day. The longer test dose procedure has been done in hundreds of hospitals in many countries, and has been able to safely prove allergy status.

If you are randomly selected to go into the **“no treatment” or control group,** you will receive a placebo capsule for 5 days which does not include penicillin. A single dose of penicillin only is used in many countries to prove or disprove penicillin allergy.

Regardless of the group you are in, the capsules will look the same. In the **“no treatment”** or **control group** this will be a **“placebo”** which does not contain penicillin. In the **“treatment”** or intervention group this will be amoxicillin 500mg. The doctors looking after you will not know which group you have been allocated to.

Participants in both groups will be followed up on day 1, 5 and 14 post testing with a short telephone call. You will also be asked to complete a questionnaire about your allergy 30 days and 90 days after you start.

There are no additional costs associated with participating in this research project, nor will you be paid. All capsules, tests and medical care done as part of the research project will be provided to you free of charge. If you decide to participate in this research project, the study doctor will inform your local doctor of the results.

**4 What do I have to do?**

You do not have to do anything in particular or stop anything you might be doing. You can still take most regular medications and will not need to change your lifestyle in other ways. You can still donate blood if you want to and take part in other studies.

**5 Other information about the research project**

We estimate that 120 participants will be taking part in this research. There are four Australian Hospitals involved (Austin Health, Peter MacCallum Cancer Centre, St George, Royal Brisbane and Womens Hospital), one Canadian center (McGill University Health Centre (MUHC), one South African centre: Groote Schuur Hospital (Capetown, SA) and one Danish hospital: Herlev and Gentofte Hospital (Copenhagen, Denmark).

**6 Do I have to take part in this research project?**

Participation in any research project is voluntary. If you do not wish to take part, you do not have to. If you decide to take part and later change your mind, you are free to withdraw from the project at any stage. If you do decide to take part, you will be given this *Participant Information and Consent Form* to sign and you will be given a copy to keep. Your decision to take part or not, or to take part and then withdraw, will not affect your routine treatment, your relationship with those treating you or your relationship with *[Study Site].*

**7 What are the alternatives to participation?**

You do not have to take part in this research project to receive treatment at this hospital. Other options are available; these include receiving a single dose challenge or prolonged challenge depending on the local site practice. The study doctor will discuss these options with you before you decide whether you can take part in this research project. You can also discuss the options with your local doctor.

**8 What are the possible benefits of taking part?**

We cannot guarantee or promise that you will receive any benefits from this research; however, possible benefits may include not requiring additional testing prior to the drug challenge and avoiding multiple dose provocation.

**9 What are the possible risks and disadvantages of taking part?**

Medical treatments often cause side effects. You may have none, some or all of the effects listed below, and they may be mild, moderate or severe. If you have any of these side effects, or you are worried about them, talk with study doctor. The study doctor will also be looking out for side effects. There may be side effects that the researchers do not expect or do not know about and that may be serious. Tell the study doctor immediately about any new or unusual symptoms.

Many side effects go away shortly after treatment ends. However, sometimes side effects can be serious, long lasting or permanent. If a severe side effect or reaction occurs, your doctor may need to stop the treatment. The doctor will discuss with you the best way of managing any side effects.

The types of side effects include allergic reactions such as mild rash (i.e. 2 in 100) or anaphylaxis (i.e.1 in 10,000). Anaphylaxis is a serious allergic response, which usually involves more than one system in the body. Symptoms such as hives, swelling and trouble breathing usually begin 5 - 30 minutes after exposure to an allergen and may lead to anaphylactic shock which can be fatal if not treated immediately. Serious reactions such as anaphylaxis have not been reported with this test dose procedure but are theoretically possible. Other side effects such as itch, nausea, vomiting, diarrhea, abnormalities of liver or kidney tests are also possible, although very unlikely.

**10 What if new information arises during this research project?**

Sometimes during the course of a research project, new information becomes available about the treatment that is being studied. If this happens, your study doctor will tell you about it and discuss whether you want to continue to participate in the research project. If you decide to withdraw, your study doctor will plan for your regular health care to continue. If you decide that you can continue in the research project you will be asked to sign an updated consent form.

If new information does become available, your study doctor might consider it to be best for you to stop the research project. If this happens, the doctor will explain the reasons and arrange for your regular health care to continue.

**11 Can I have other treatments during this research project?**

You may not be able to take some or all of the medications or treatments you have been taking for your condition or for other reasons. It is important to tell your study doctor and the study staff about any treatments or medications that you may be taking, including over-the-counter medications, vitamins or herbal remedies, acupuncture or other alternative treatments. You should also tell your study doctor about any changes to these during your participation in the study. Your study doctor will also explain which treatments or medications you may need to stop when you are in the study.

**12 What if I withdraw from this research project?**

If you decide to withdraw from the study, please let one of research team know. This means that they can discuss any health risks or special requirements that may come with withdrawing.

If you do withdraw during the study, the study doctor and relevant study staff will not collect additional personal information from you, although personal information already collected will be retained to ensure that the results of the research project can be measured properly and to comply with law. You should be aware that data collected by the researchers up to the time you withdraw will be part of the research project results. If you do agree with this, you must tell the research team before you join the research project.

**13 Could this research project be stopped unexpectedly?**

This research project may be stopped unexpectedly for a variety of reasons. These may include reasons such as:

• Unacceptable side effects;

• The drug/treatment/device being shown not to be effective;

• The drug/treatment/device being shown to work and not need further testing;

• Decisions made in the commercial interests of the sponsor or by local regulatory/health authorities.

**14 What happens when the research project ends?**

When the study is finished, a report will be completed and published in the scientific literature. If you would like a report of the findings, please let one of the research team know.

**Part 2 How is the research project being conducted?**

**15 What will happen to the information about me?**

By signing the consent form you agree to the study doctor and relevant research staff collecting and using personal information about you for the research. Any information that can identify you will remain confidential. Your information will be entered into a secure database as a study number with no personally identifiable information. Only study investigators will have access to it. Your information will only be used for the purpose of this research project and it will only be disclosed with your permission, except as required by law.

Information about you may be obtained from your health records held at this hospital and other health services for the purpose of this research. By signing the consent form you agree to the study team accessing health records if they are relevant to participation in this research project. Your health records and any information collected and stored by the study doctor during the research project may be reviewed for the purpose of verifying the procedures and the data. By signing the Consent Form, you give permission for the release of, or access to, this confidential information to the study team.

We expect that the results of this research project will be published and or presented in a number of ways. In any publication and/or presentation, information will be provided in such a way that you cannot be identified, except with your permission. No identifying information about the people who took part will be included. Information about participation in this research project may be recorded in your health records.

In accordance with relevant Australian and/or Victorian privacy and other relevant laws, you have the right to request access to the information collected and stored by the study team. You also have the right to request that any information with which you disagree be corrected. Please contact the study team member named at the end of this document if you would like to access this information.

**16 Who is organising and funding the research?**

This research project is being conducted by Prof Jason Trubiano of Austin Health. In addition, if knowledge leant through this research leads to discoveries that are of commercial value to the study doctors or their institutions, there will be no financial benefit to you or your family from these discoveries. No member of the research team will receive a personal financial benefit from your involvement in this research project (other than their ordinary wages).

**17 Who has reviewed the research project?**

All research in Australia involving humans is reviewed by an independent group of people called a Human Research Ethics Committee (HREC). The ethical aspects of this research project have been approved by the HREC of Austin Health. This project will be carried out according to the *National Statement on Ethical Conduct in Human Research (2007)*. This statement has been developed to protect the interests of people who agree to participate in human research studies.

**18 Further information and who to contact**

The person you may need to contact will depend on the nature of your query.

If you want any further information concerning this project or if you have any medical problems that may be related to your involvement in the project (for example, any side effects), you can contact the principal study doctor, *[Site Study Doctor]* at [*[contact*](mailto:antibiotic.allergy@austin.org.au) *details],* quoting “PROSPECTOR Study” in the email title or any of the following people:

**Clinical contact person**

| Name | *[Name]* |
| --- | --- |
| Position | *[Position]* |
| Telephone | *[Contact number]* |
| Email | *[Email]* |

For matters relating to research at the site at which you are participating, the details of the local site complaints person are:

**Complaints contact person**

| Position | *[Position]* |
| --- | --- |
| Telephone | *[Contact Number]* |
| Email | *[Email]* |

If you have any complaints about any aspect of the project, the way it is being conducted or any questions about being a research participant in general, then you may contact:

| Reviewing HREC name | Austin Health Human Research Ethics Committee |
| --- | --- |
| HREC Executive Officer | Manager, Office for Research |
| Telephone | +61 3 9496 4035 |
| Email | [research@austin.org.au](mailto:ethics@austin.org.au) |

**Reviewing HREC approving this research** **and HREC Executive Officer details**

**Consent Form**

| **Title** | Prolonged versus single dose in penicillin oral challenge testing randomized control trial – PROSPECTOR Study |
| --- | --- |
| **Short Title** | PROSPECTOR Study |
| **Protocol Number** | PROSPECTOR Version 6 dated 16 July 2023 |
| **Coordinating Principal Investigator** | Professor Jason A Trubiano |
| **Site Principal Investigator** | *[Site Principal Investigator]* |
| **Associate Investigator(s)** | *[Associate Investigators]* |
| **Study Site** | *[Study Site]* |

**Declaration by Participant**

I have read the *Participant Information Sheet* or someone has read it to me in a language that I understand.

I understand the purposes, procedures and risks of the research described in the project.

I have had an opportunity to ask questions and I am satisfied with the answers that I have received.

I believe that my participation in this study is not contrary to my best interests/my preferences and values and my social wellbeing.

I freely agree to participate in this research project as described and understand that I am free to withdraw at any time during the project without affecting my future health care.

I understand that I will be given a signed copy of this document to keep.

I give permission for my doctors, other health professionals, hospitals or laboratories outside this hospital to release information to Austin Health concerning my condition and treatment for the purposes of this project. I understand that such information will remain confidential.

| Name of Participant (please print) _______________________________________________________________  Signature ______________________________________ Date ___________________________ |
| --- |

| Declaration - for participants unable to read the information and consent form  Witness to the informed consent process*  Name (please print) _____________________________________________________________  Signature _____________________________________ Date ____________________________  *Witness is not to be the Investigator, a member of the study team or their delegate. Witness must be 18 years or older. |
| --- |

**Declaration by Study Doctor/Senior Researcher^†^**

I have given a verbal explanation of the research project; its procedures and risks and I believe that the participant has understood that explanation.

|  | | | | | | | |
| --- | --- | --- | --- | --- | --- | --- | --- |
|  | Name of Study Doctor/  Senior Researcher^†^ (please print) | |  | | |  | |
|  | | | | | |  | |
|  | Signature |  | | Date |  | |  |
|  | | | | | | | |

^†^ A senior member of the research team must provide the explanation of, and information concerning, the research project.

Note: All parties signing the consent section must date their own signature.

**Form for Withdrawal of Participation**

| **Title** | Prolonged versus single dose in penicillin oral challenge testing randomized control trial – PROSPECTOR Study |
| --- | --- |
| **Short Title** | PROSPECTOR Study |
| **Protocol Number** | PROSPECTOR Version 6 dated 16 July 2023 |
| **Coordinating Principal Investigator** | Professor Jason A Trubiano |
| **Site Principal Investigator** | *[Site Principal Investigator]* |
| **Associate Investigator(s)** | *[Associate Investigators]* |
| **Study Site** | *[Study Site]* |

**Declaration by Participant**

I wish to withdraw from participating in the above research project and understand that this withdrawal will not affect my routine treatment, my relationship with the medical staff or with the treating hospital.

| Name of Participant (please print) _______________________________________________________________  Signature ______________________________________ Date ___________________________ |
| --- |

**Declaration by Study Doctor/Senior Researcher^†^**

I have given a verbal explanation of the implications of withdrawal from the research project and I believe that the participant has understood that explanation.

|  | | | | | | | |
| --- | --- | --- | --- | --- | --- | --- | --- |
|  | Name of Study Doctor/  Senior Researcher^†^ (please print) | |  | | |  | |
|  | | | | | |  | |
|  | Signature |  | | Date |  | |  |
|  | | | | | | | |

^†^ A senior member of the research team must provide the explanation of, and information concerning, the research project.

Note: All parties signing the consent section must date their own signature

**Supplementary Material 2.** Patient Information and Consent Form for PROSPECTOR-2

# Participant Information and Consent Form

| **Short Name of Project** | PROSPECTOR-2 |
| --- | --- |
| **Full Name of Project** | PROlonged versus Single dose in PEnicillin oral Challenge Testing double-blind parallel randomized placebo cOntrolled tRial |
| **Coordinating Principal Investigator** | Professor Jason Trubiano |
| **Site Principal Investigator** | *[Site Principal Investigator]* |
| **Site Name** | *[Name of site]* |


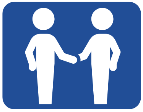


### What am I being invited to do?

We invite you to take part in a project that is looking at whether a single dose of penicillin or a longer course is needed to work out if someone is allergic to penicillin. You have been invited to take part because you have had either a delayed onset allergy to penicillin or an allergy of unknown timing after receiving a single dose of amoxycillin as a test dose in an oral challenge.

Around 830 people will take part in this project. They will be from hospitals around Australia and overseas.

Please read this information and feel free to ask any questions. You can take some time to make up your mind and decide if this project is right for you. You can also talk to someone you trust, like a family member, friend, or your local doctor.


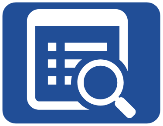


### What is the purpose of this project?

In this project, we will ask you about your allergy and medical history and give you a single dose of amoxicillin (with or without prior skin testing (which take approximately 30 minutes)) to see if you develop a reaction. If you do, you will be given the standard treatment by the doctor looking after you. If you don’t develop a reaction, you will then be able to take part in the study if you would like to. If you decide to take part, you will be randomly assigned to one of these two groups:

**Group 1: Penicillin**

5 days of amoxicillin capsules twice a day

**Group 2: Placebo**

5 days of placebo* capsules twice a day with no amoxicillin

**The placebo looks the same as an amoxicillin capsules but will not contain amoxicillin. It is a sugar-filled capsule.*

Neither you or your study doctor will know if you have the placebo or amoxicillin as both are in the same capsule form. At the end of the study period, there is a possibility that you will be unblinded (if deemed appropriate by the site principal investigator).

Upon discharge, you will be supplied with a prescription for oral corticosteroids and antihistamines to be used in the setting of an immune-mediated positive oral challenge. A site investigator will instruct you to fill this script at your own expense if required.

We have already completed a small study to see if this approach is safe.

If you decide you want to take part in the research project, you will be asked to sign the consent section. By signing it you are telling us that you:

• Understand what you have read;

• Consent to taking part in the research project;

• Consent to having the tests and treatments that are described;

• Consent to the use of your personal and health information as described.

You will be given a copy of this *Participant Information and Consent Form* to keep.


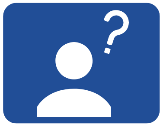


### Do I have to take part and can I change my mind?

**Taking part is up to you**

You can decide whether you take part in this project. You can say no if you want to.

Your decision won’t affect your relationship with your doctor or *[Study Site]*.

**You can change your mind at any time**

If you do take part, you can stop at any time. If you want to stop, please tell someone in the project team. You do not have to tell us the reason.

Once you stop taking part, we will not collect any more information about you. We will keep the information we have already collected to make sure the results of the project can be measured properly.

**The project might stop for other reasons**

We might need to stop the project while you are taking part. If this happens, we will explain the reasons to you.

We may also ask you to stop taking part in the project if it is no longer in your best interest. If this happens, we will discuss this with you.


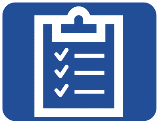


### What do I have to do if I take part?

If you take part in this project, you will be in it for four months.

This table below outlines what you need to do in this project. For more information, please ask a member of the project team.

| **What part of the project?** | **What do I have to do?** |
| --- | --- |
| Consenting to take part in this project | If you are happy to take part in this project, you will be asked to sign a consent form |
| During the project | For the first five days you will take the capsules twice a day  We will contact you by telephone/telehealth four times during the study – on days 1, 5, 7 and 14 to complete a short electronic questionnaire.  You will be asked to complete a questionnaire on day 30, 90 and 120 via email/telephone.  We will ask you to complete a questionnaire before you start the study and again on day 90. This will take about 5 minutes |

By taking part, you will help the researchers understand more about penicillin allergies. This knowledge may help people in the future.

You may not directly benefit from taking part in this project. It is possible but unknown whether this study will help determine what testing is required to accurately diagnose penicillin allergies.

### If I take part, what are the possible risk?

Medical treatments often cause side effects. You may have none, some or all of the effects listed below, and they may be mild, moderate or severe. If you have any of these side effects, or you are worried about them, talk with study doctor. The study doctor will also be looking out for side effects. There may be side effects that the researchers do not expect or do not know about and that may be serious. Tell the study doctor immediately about any new or unusual symptoms.

Many side effects go away shortly after treatment ends. However, sometimes side effects can be serious, long lasting or permanent. If a severe side effect or reaction occurs, your doctor may need to stop the treatment. The doctor will discuss with you the best way of managing any side effects.

The types of side effects include allergic reactions such as mild rash (i.e. 2 in 100) or anaphylaxis (i.e.1 in 10,000). Anaphylaxis is a serious allergic response, which usually involves more than one system in the body. Symptoms such as hives, swelling and trouble breathing usually begin 5 - 30 minutes after exposure to an allergen and may lead to anaphylactic shock which can be fatal if not treated immediately. Serious reactions such as anaphylaxis have not been reported with this test dose procedure but are theoretically possible. Other side effects such as itch, nausea, vomiting, diarrhea, abnormalities of liver or kidney tests are also possible, although very unlikely.


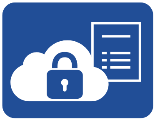


### If I take part, what will happen to my information and samples?

**Collecting your information**

We will collect information for the project from your medical records and directly from you.

**Keeping your information safe**

To keep your information safe, we will:

follow all relevant privacy requirements

keep it securely on an electronic database (University of Melbourne REDCap)

take steps to prevent anyone from accessing information that identifies you unless they need to, for example, to check it in an audit

give it a code and keep it separate from anything that could easily identify you, like your name or contact information.

You can ask us to tell you what information we have collected about you as part of this project. If your information is not correct, you can also ask us to change it.

We will keep your information for 15 years. After this, we will destroy it in accordance with hospital policy.

**Sharing your information with others**

We will share some of your information with others.

**Sharing information with other researchers:** we will share certain information from this project so that other researchers can use it in the future. These researchers may be in Australia or overseas. We will only share information that has been de-identified, no personal identifiable information will be shared.

**Getting more information**

If you would like to know more about how we will collect, store and share your information as part of this project, please ask one of the research team.


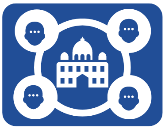


### Who is running and paying for this project?

This project is being run by Prof Jason Trubiano of Austin Health. In addition, if knowledge leant through this research leads to discoveries that are of commercial value to the study doctors or their institutions, there will be no financial benefit to you or your family from these discoveries. No member of the research team will receive a personal financial benefit from your involvement in this research project (other than their ordinary wages).


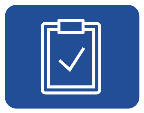


### Who has approved this project?

The Austin Health Human Research Ethics Committee has approved this project. This committee makes sure that this project meets Australian ethical standards for research that involves people.

**Complaints about how this project is being run**

If you have any complaints about how this project is being run, please contact:

Name: Manager, Discovery and Innovation Unit

Contact details: Tel: (03) 9496 4090 Email: [feedback@austin.org.au](mailto:feedback@austin.org.au)


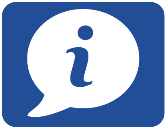


### Where can I find more information?

Thank you for taking the time to read this information about our project. The person you may need to contact will depend on the nature of your query. If you want any further information concerning this project or if you have any medical problems which may be related to your involvement in the project (for example, any side effects), You can contact a member of the project team at any time to ask questions.

*Name Role Contact details (phone number preferred)*

*Name Role Contact details (phone number preferred)*

# Signature Page

| **Short Name of Project** | PROSPECTOR-2 |
| --- | --- |
| **Full Name of Project** | PROlonged versus Single dose in PEnicillin oral Challenge Testing double-blind parallel randomized placebo cOntrolled tRial |
| **Coordinating Principal Investigator** | Professor Jason Trubiano |
| **Site Principal Investigator** | *[Site Principal Investigator]* |
| **Site Name** | *[Name of site]* |

| **Consent to take part in this project** |
| --- |
| By signing this consent form, I acknowledge that:  I freely agree to take part in this project  I understand that I can stop taking part in the project at any time  I have read, or have had read to me, the information provided about this project and understand what is involved  I have had the opportunity to consider the information, ask questions and am satisfied with the answers I received  I give permission for my medical records to be accessed for the purposes of this project |

**Person taking part in the project**

Signature: ______________________________________________ Date: ______________

Name: _________________________________________________

**Person conducting the informed consent discussion**

I have explained the research project, its procedures and risks to the participant and I believe they have understood that explanation.

Signature: ______________________________________________ Date: ______________

Name: __________________________________

Each person must sign and personally date this consent form

# Form of Withdrawal of Participation

| **Short Name of Project** | PROSPECTOR-2 |
| --- | --- |
| **Full Name of Project** | PROlonged versus Single dose in PEnicillin oral Challenge Testing double-blind parallel randomized placebo cOntrolled tRial |
| **Coordinating Principal Investigator** | Professor Jason Trubiano |
| **Site Principal Investigator** | *[Site Principal Investigator]* |
| **Site Name** | *[Name of site]* |

**Declaration by Participant**

I wish to withdraw from participating in the above research project and understand that this withdrawal will not affect my routine treatment, my relationship with the medical staff or with the treating hospital.

Signature: ______________________________________________ Date: ______________

Name: _________________________________________________

**Declaration by Study Doctor/Senior Researcher†**

I have given a verbal explanation of the implications of withdrawal from the research project and I believe that the participant has understood that explanation.

Signature: ______________________________________________ Date: ______________

Name: __________________________________

Each person must sign and personally date this withdrawal of participation form
