# Supplementary material for: Prolonged versus single dose in penicillin oral challenge testing: protocols for a pilot and definitive randomised controlled trial (PROSPECTOR studies)
Source: BMJ Open. 2025 Feb 22;15(2):e094712. doi: 10.1136/bmjopen-2024-094712 (PMC11848670; doi:10.1136/bmjopen-2024-094712)
Supplement: Supplementary file 2 [file bmjopen-2024-094712.reviewer_comments.pdf]

PEER REVIEW HISTORY

BMJ Open publishes all reviews undertaken for accepted manuscripts. Reviewers are asked to complete a checklist review form and are provided with free text boxes to elaborate on their assessment. These free text comments are reproduced below.

ARTICLE DETAILS

Title (Provisional)

Prolonged versus single dose in penicillin oral challenge testing – Protocols for a pilot and definitive randomised controlled trial (PROSPECTOR Studies)

Authors

Ng, Irvin; James, Fiona; Copaescu, Ana; Vogrin, Sara; Mitri, Elise; Rose , Morgan; Sullivan, Richard; Lane, Michael; Legg, Amy; Godsell, Jack; Fernando, Suran; Garvey, Lene Heise; Sabato, Vito; Li, Philip; Peter, Jonathan Grant; Trubiano, Jason

---

VERSION 1 - REVIEW

**Reviewer**	**1**
**Name**	**Powell, Neil**
**Affiliation**	**Royal Cornwall Hospitals NHS Trust**
**Date**	**13-Dec-2024**
**COI**	**NIHR funding for penicillin allergy doctoral fellowship**

Co-applicant on NIHR funded SPACE study
Principal investigator NIHR funded ALABAMA study.
UK co-ordinator for iNAAN study

This study addresses an important gap in penicillin allergy de-labelling knowledge and aims to capture both the potential increased sensitivity of prolonged challenges in identifying penicillin allergy as well as the potential harms. some minor comments form me for consideration;

page 9 line 3 - says amoxicillin will be the prolonged challenge drug. Is that all patients, or only those where the index penicillin is unknown. I.e. index penicillin, where known, or amoxicillin if unknown.

page 10 line 14 - please somewhere define 'specialist allergy healthcare provider'

page 11 line - line 8 how will acquisition of MDR organism be measured? clinical samples collected during routine clinical care or will patients be screened / swabbed?

Page 15 line 56 - the sentence ends abruptly. ?missing info here?

Page 16 line 6 - interesting approach to give prescription in case of need. Is this to reduce any burden on primary care services? it seems like a lot of prescription generation when in reality very few, if any, will be required.

**Reviewer**	**2**
**Name**	**Maraolo, Alberto**
**Affiliation**	**Cotugno Hospital**
**Date**	**21-Dec-2024**
**COI**	**None**

Very interesting project.

Some criticisms.

1) Too many references in the introduction.

2) Please better clarify if people with type-1 reactions are excluded. Just subjects with type-2/3/4 are included, right?

3) Exclusion criterion number 2 is too generic and broad.

4) Unclear aspect: it seems that only people with low PEN-FAST score are included, but in the subgroup analyses PEN-FAST score >3 patients are considered.

5) Confidence interval width below 20% for any proportion in both direction? For instance 0.8-1.2? Or to be added (0.9-1.1)?

VERSION 1 - AUTHOR RESPONSE

Response to reviewer 1:

- **page 9 line 3 - says amoxicillin will be the prolonged challenge drug. Is that all patients, or only those where the index penicillin is unknown. I.e. index penicillin, where known, or amoxicillin if unknown.**

***Response*:** We thank the reviewer for this comment. All participants who are randomized to the intervention arm will receive amoxicillin 500 mg BD for 5 days. We have clarified this sentence to “Participants randomised to the intervention arm will receive a prolonged, 5-day course of oral 500mg amoxicillin, administered twice daily, to commence the day following the initial single-dose amoxicillin oral challenge” [page 9 line 2].

- **page 10 line 14 - please somewhere define 'specialist allergy healthcare provider'**

***Response*:** We thank the reviewer for this suggestion and have now included a definition on specialist allergy healthcare provider [page 10 line 21-22].

- **page 11 line - line 8 how will acquisition of MDR organism be measured? clinical samples collected during routine clinical care or will patients be screened / swabbed?**

***Response*:** We thank the reviewer for this comment. Acquisition of MDR organism will be measured through data collected during routine clinical care. This information will be obtained through email/telephone follow-up questionnaire administered at days 30, 90 and 120, as well as a review of the patient's medical record.

- **Page 15 line 56 - the sentence ends abruptly. ?missing info here?**

***Response*:** Thank you for this comment. This was an oversight, as the sentence had initially been removed during drafting but was inadvertently left incomplete in the manuscript. We have corrected this in the revised version.

- **Page 16 line 6 - interesting approach to give prescription in case of need. Is this to reduce any burden on primary care services? it seems like a lot of prescription generation when in reality very few, if any, will be required.**

***Response*:** Thank you for this response. As a safety and feasibility trial this was instigated as a safety mechanism. For the definitive PROSPECTOR-2 study due to the ease of administration and saving of primary care intervention and prompt action this was deployed again.

<u>**Response to reviewer 2:**</u>

- **Too many references in the introduction.**

***Response*:** Thank you for the comment, we have now reduced the number of references in the introduction . In addition, we have noted some formatting error with our references, which in-text references have been previously deleted but still listed in the reference section of the manuscript. This has now been rectified

- **Please better clarify if people with type-1 reactions are excluded. Just subjects with type-2/3/4 are included, right?**

***Response*:** Thank you. Type 1 reactions are excluded. Any patient that passes the direct oral amoxicillin challenge can be recruited if they have a delayed or unknown timing **reaction. We see no significant benefit in giving prolonged oral challenges in patients with a clear single dose history of Type 1/IgE mediated hypersensitivity.**

- **Exclusion criterion number 2 is too generic and broad.**

***Response*:** Thank you for this comment. This exclusion criteria has been utilised in our two previous randomised control trials. Whilst certainly broad we did not see a large number of patients excluded in our previous RCTs (Palace and ORACLE) hence its retention

- **Unclear aspect: it seems that only people with low PEN-FAST score are included, but in the subgroup analyses PEN-FAST score >3 patients are considered.**

***Response*:** We thank the reviewer for this comment and have provided clarification on page 8 line 16-17. Participant with PEN-FAST score of ≥3 are eligible for inclusion in the study provided that they meet the broader inclusion/exclusion criteria.

- **Confidence interval width below 20% for any proportion in both direction? For instance 0.8-1.2? Or to be added (0.9-1.1)?**

***Response*:** Thank you for your question. The confidence interval (CI) width of 20% refers to the entire width of the interval, rather than the deviation in each directions. For example, if the proportion is 30%, a CI with a width of 20% would range from approximately 20% to 40%. As the reviewer has noted, the CI may not be symmetrical around the point estimate. However, the total width of the interval remains 20%. Response to reviewers' comments

---

VERSION 2 - REVIEW

**Reviewer**	**2**
**Name**	**Maraolo, Alberto**
**Affiliation**	**Cotugno Hospital**
**Date**	**31-Jan-2025**
**COI**	

The authors replied to all the comments.
